# Early life stress and serotonin transporter gene variation interact to affect the transcription of the glucocorticoid and mineralocorticoid receptors, and the co-chaperone FKBP5, in the adult rat brain

**DOI:** 10.3389/fnbeh.2014.00355

**Published:** 2014-10-13

**Authors:** Rick H. A. van der Doelen, Francesca Calabrese, Gianluigi Guidotti, Bram Geenen, Marco A. Riva, Tamás Kozicz, Judith R. Homberg

**Affiliations:** ^1^Department of Anatomy, Donders Institute for Brain, Cognition and Behaviour, Radboud University Medical CenterNijmegen, Netherlands; ^2^Department of Cognitive Neuroscience, Donders Institute for Brain, Cognition and Behaviour, Radboud University Medical CenterNijmegen, Netherlands; ^3^Department of Pharmacological and Biomolecular Sciences, Università degli Studi di MilanoMilan, Italy

**Keywords:** early life stress, serotonin transporter, depression, glucocorticoid receptor, mineralocorticoid receptor, FKBP5, hippocampus, medial prefrontal cortex

## Abstract

The short allelic variant of the serotonin transporter (5-HTT) promoter-linked polymorphic region (5-HTTLPR) has been associated with the etiology of major depression by interaction with early life stress (ELS). A frequently observed endophenotype in depression is the abnormal regulation of levels of stress hormones such as glucocorticoids. It is hypothesized that altered central glucocorticoid influence on stress-related behavior and memory processes could underlie the depressogenic interaction of 5-HTTLPR and ELS. One possible mechanism could be the altered expression of the genes encoding the glucocorticoid and mineralocorticoid receptors (GR, MR) and their inhibitory regulator FK506-binding protein 51 (FKBP5) in stress-related forebrain areas. To test this notion, we exposed heterozygous (5-HTT^+/−^) and homozygous (5-HTT^−/−^) serotonin transporter knockout rats and their wildtype littermates (5-HTT^+/+^) to daily 3 h maternal separations from postnatal day 2 to 14. In the medial prefrontal cortex (mPFC) and hippocampus of the adult male offspring, we found that GR, MR, and FKBP5 mRNA levels were affected by ELS × 5-HTT genotype interaction. Specifically, 5-HTT^+/+^ rats exposed to ELS showed decreased GR and FKBP5 mRNA in the dorsal and ventral mPFC, respectively. In contrast, 5-HTT^+/−^ rats showed increased MR mRNA levels in the hippocampus and 5-HTT^−/−^ rats showed increased FKBP5 mRNA in the ventral mPFC after ELS exposure. These findings indicate that 5-HTT genotype determines the specific adaptation of GR, MR, and FKBP5 expression in response to early life adversity. Therefore, altered extra-hypothalamic glucocorticoid signaling should be considered to play a role in the depressogenic interaction of ELS and 5-HTTLPR.

## Introduction

Vulnerability to stress-related psychiatric disease is determined by a complex interplay of genome and environment. The moderation of the effects of stressful life events by the serotonin transporter (5-HTT) gene-linked polymorphic region (5-HTTLPR) is a well-known example of such a gene × environment (G×E) interaction (Caspi et al., 2003). Specifically, the short (S) allele of 5-HTTLPR has been associated with a significantly increased risk to develop depression in interaction with adverse events such as childhood abuse (Karg et al., 2011, but also see Risch et al., 2009). Compared to L/L homozygotes, individuals with the S-allele have an approximate two-fold reduction in the promoter activity of the 5-HTT gene (Heils et al., 1996; Lesch et al., 1996; Greenberg et al., 1999). The consequences of lower 5-HTT availability can be studied by use of homozygous and heterozygous 5-HTT knockout (5-HTT^−/−^, 5-HTT^+/−^) rodents. The 5-HTT^+/−^ rodents are considered to be the superior genotype model of the S-allele, as they display a two-fold reduction in 5-HTT availability (Bengel et al., 1998; Homberg et al., 2007), and increased sensitivity to stressful events (Carola et al., 2008; Narayanan et al., 2011; van der Doelen et al., 2013), just as 5-HTTLPR S-allele carriers. Yet, at baseline, 5-HTT^−/−^ rodents show superior behavioral similarity with S-allele healthy controls, as expressed by anxiety- and depressive-like behavior (Holmes et al., 2003; Lira et al., 2003; Olivier et al., 2008; Schipper et al., 2011). Therefore, it has been argued that both 5-HTT^+/−^ and 5-HTT^−/−^ rodents are useful to study the underlying biology of ELS × 5-HTTLPR interaction (Caspi et al., 2010; Kalueff et al., 2010; Homberg and van den Hove, 2012).

One of the candidate biological systems that has been linked to this GxE interaction is the stress-responsive hypothalamo-pituitary-adrenal (HPA) axis. A stress response of the HPA-axis is initiated by parvocellular neurons in the paraventricular nucleus (PVN) of the hypothalamus, by secreting corticotropin-releasing factor (CRF) at the median eminence to stimulate the anterior pituitary to synthesize and release adrenocorticotropic hormone (ACTH), which itself stimulates the synthesis and release of glucocorticoids (cortisol in humans, corticosterone in rodents) from the adrenal cortex. The HPA-axis is regulated through direct feedback action of glucocorticoids at the level of the pituitary and the PVN, but importantly also by extra-hypothalamic brain areas such as the medial prefrontal cortex (mPFC), hippocampus and extended amygdala (Ulrich-Lai and Herman, 2009). This feedback action is mediated via the mineralocorticoid and glucocorticoid receptors (MR, GR), with MR mainly involved in maintaining basal HPA activity and GR with recovery from stress-induced activity. Glucocorticoids furthermore act via these receptors on peripheral tissues as well as the brain to affect physiology and behavior, and to facilitate an integrative stress response (De Kloet et al., 1998, 2005; Champagne et al., 2009).

In depression, about 50% of patients display hyperactivity of the HPA-axis as represented by basal hypercortisolemia and resistance to GR-mediated suppression of glucocorticoid levels (Checkley, 1996; Pariante and Miller, 2001). In contrast, HPA hypoactivity has been observed for atypical depression and post-traumatic stress disorder, the latter being associated with increased GR sensitivity (Gold and Chrousos, 2002; Yehuda, 2009). Further, single nucleotide polymorphisms and expression levels of the genes encoding GR (*NR3C1*) and MR (*NR3C2*) have been associated with depression (Webster et al., 2002; van Rossum et al., 2006; Klok et al., 2011a,b; Medina et al., 2013; Qi et al., 2013). Specifically, in major depression postmortem studies have documented decreased hippocampal GR and MR mRNA levels (Webster et al., 2002; Klok et al., 2011a). The decrease in hippocampal MR expression in major depression could be restricted to the anterior hippocampus (Medina et al., 2013). Furthermore, lower MR mRNA levels in different areas of the prefrontal cortex have been reported for depressed compared to non-depressed subjects (Klok et al., 2011a; Qi et al., 2013). Therefore, there is convincing evidence that altered glucocorticoid signaling through altered expression of GR and MR in forebrain areas is highly relevant in the pathophysiology of depression (Holsboer, 2000).

Recently, FK506-binding protein 51 (FKBP5), a co-chaperone of steroid hormone receptors, has emerged as an important regulator of stress-induced GR-mediated effects (Binder, 2009). Genetic variation of *FKBP5* has additionally been shown to interact with early life stress (ELS) to epigenetically program GR-induced transcription of *FKBP5*, leading to increased risk for the development of stress-related psychiatric disorders (Klengel et al., 2013). Furthermore, the expression levels of *Fkbp5* and *Nr3c1* in the adult rat brain have recently been reported to be sensitive to chronic stress (CS) exposure and antidepressant treatment (Guidotti et al., 2013), while *Fkbp5* knockout mice seem to be less vulnerable to CS exposure (Hartmann et al., 2012). In addition, CS has been shown to lead to a disruption of the extra-hypothalamic control of HPA function (Radley et al., 2013).

Altogether, altered central glucocorticoid signaling is a plausible contributing factor to the increased vulnerability of childhood trauma-exposed 5-HTTLPR S-allele carriers to psychopathology. Previously, we have shown that our animal model of ELS × 5-HTT genotype interaction displays differential susceptibility to inescapable stress and altered activity of the HPA-axis (van der Doelen et al., 2013, 2014). In the HPA-axis, we predominantly found G×E programming of the adrenal gland, while gene expression in the pituitary and PVN was largely unaffected (van der Doelen et al., 2014). Therefore, we hypothesized that if there are adaptations in the expression of GR, MR, and/or FKBP5 in our animal model of ELS × 5-HTT genotype interaction, these adaptations would take place in extra-hypothalamic brain regions. To test this hypothesis we exposed 5-HTT heterozygous (5-HTT^+/−^) and homozygous (5-HTT^−/−^) knockout rats to ELS, i.e., maternal separation (MS), and examined the expression of GR, MR, and FKBP5 in the mPFC, hippocampus, amygdala, and bed nucleus of the stria terminalis (BNST). In addition to their regulatory function of the HPA-axis (Ulrich-Lai and Herman, 2009), these brain areas are known for their involvement in stress-related behavioral processes such as cognitive control, learning and memory, and fear and anxiety output (De Kloet et al., 1998, 2005; LeDoux, 2000; Amat et al., 2005; Kim et al., 2013).

## Materials and methods

### Animals

The experiments were approved by the Committee for Animal Experiments of the Radboud University Nijmegen, The Netherlands, and all efforts were made to minimize animal suffering, to reduce the number of animals and to utilize alternatives to *in vivo* techniques. Serotonin transporter knockout rats (Slc6a4^1Hubr^) were generated by N-ethyl-N-nitrosurea (ENU)-induced mutagenesis (Smits et al., 2006). Experimental animals (5-HTT^−/−^, 5-HTT^+/−^, and 5-HTT^+/+^ rats) were derived from crossing 3 month old 5-HTT^+/−^ rats that were outcrossed for at least 12 generations with commercial (Harlan, Ter Horst, The Netherlands) wild-type Wistar rats. The pregnant dams were housed in standard polypropylene cages (40 × 20 × 18 cm) with sawdust bedding and *ad libitum* access to water and rodent chow (Sniff Spezialdiäten, Soest, Germany) in a temperature (21 ± 1°C) and humidity-controlled room (45–60% relative humidity), with a 12:12 h light: dark cycle (lights on at 07.00 a.m.). The dams were inspected daily for delivery at 5.00 p.m. and day of birth was designated as postnatal day (PND) 0. At PND1, two paper towels (22.5 × 24.5 cm) were supplied to the mother for nest construction. Further, the litters (l) were culled to a maximum of 10 pups (1 l had only 9 pups, another only 8 pups), with gender ratios in favor of a male majority (5:5 to maximally 7:3).

### Early life stress

We used repeated and prolonged MS as a model for ELS, as this paradigm has previously been shown to affect the HPA-axis functioning and stress coping behavior of the offspring (Plotsky and Meaney, 1993; Francis et al., 2002; Ladd et al., 2004; Levine, 2005; Plotsky et al., 2005; Macrì and Würbel, 2006; van der Doelen et al., 2014). Litters were randomly allocated to one of two rearing conditions (PND 2–14): MS for 180 min (MS180) or a control treatment with immediate reunion of mother and pups (MS0). MS180 was started daily between 08.30 and 09.00 a.m., and consisted of the following procedure: The mother was removed from the home cage and placed into an identical cage until the end of the separation period. Pups were then removed from the nest as complete litters and placed into a cage (24 × 15 × 14 cm) with clean sawdust bedding, and then transferred to an adjacent room. There, the cages were placed on heat pads, which were set to maintain a bedding temperature of 31–33°C for PND 2–7 and 29–31°C for PND 8–14. At the end of the separation period, litters were returned to their home cage by placing them in the nests and sprinkling soiled home cage bedding over them. This was followed by reunion with the mothers. We have previously reported that this procedure affects maternal care behavior across PND 2–8 (van der Doelen et al., 2014). During PND 0–22, half of the bedding material of the home cages was refreshed every week. At PND 14, ear punches were taken of the pups for identification and genotyping, which was performed by Kbiosciences (Hoddesdon, UK). The procedure of genotyping has been described previously (Homberg et al., 2007). At PND 22, the pups were weaned and housed in groups of 2–3 littermates of the same sex and rearing, under the same conditions as mentioned above.

### Tissue collection

For the collection of tissues only adult (PND 85–95) male rats were used. These rats were derived of 13 l that were subjected to MS180 and 12 l that received the control treatment (MS0). Of every litter, where possible, a single rat was selected of all three genotypes. The rats were sacrificed between 9.00 a.m. and 2.00 p.m. by acute decapitation. Across this time period, the rats were randomized for their genotype and early life treatment. Immediately after decapitation, the brains were isolated, frozen in aluminum foil on dry ice and stored at −80°C. In a cryostat (−15°C), the brains were prepared in 420 μm-thick coronal slices in order to obtain punches from dorsal and ventral parts (prelimbic, infralimbic, respectively) of the mPFC (Bregma +3.72 and +3.30 mm), the anterodorsal part of the BNST (Bregma +0.24 and −0.18 mm), central amygdala (Bregma −1.72 and −2.14 mm), and dorsal (Bregma −2.14 and −2.56 mm) and ventral hippocampus (Bregma −4.80 and −5.22 mm). The brain areas were bilaterally punched out with a Miltex 1.5 (hippocampal samples) or 1.0 mm (other areas) biopsy puncher (Integra Miltex, York, PA, USA), collected in sterile vials, immediately placed on dry ice and stored at −80°C. Representative images of punched sections and group sizes are available in the Supplementary Material.

### RNA isolation and gene expression analysis by quantitative real-time PCR

Total RNA was isolated by a single step of guanidinium isothiocyanate/phenol extraction using PureZol RNA isolation reagent (Bio-Rad, Hercules, CA, USA) according to manufacturer's instructions. RNA concentrations were measured and RNA purity checked (A260/280 ratio between 1.8 and 2.0) with a NanoDrop 1000 spectrophotometer (Thermo Fisher Scientific, Waltham, MA, USA). Samples were treated with DNase to avoid DNA contamination. As the study was a collaborative effort, real-time PCR (RT-PCR) was performed both in Milan (mPFC and hippocampal samples) and Nijmegen (anterodorsal BNST and central amygdala). To exclude possible differential results depending on the methods, adrenal gene expression was assessed in both labs and yielded identical statistical conclusions as previously published (van der Doelen et al., 2014). In Nijmegen first strand cDNA synthesis was performed by incubating 40 ng of RNA dissolved in 12 μl of Rnase-free water containing 0.25 mU random hexamer primers (Roche Applied Science, Penzberg, Germany) at 70°C for 10 min, followed by double-strand synthesis in 1st strand buffer with 10 mM DTT, 100 U Superscript II (Life Technologies), 0.5 mM dNTPs (Roche Applied Science) and 20 U of rRNasin (Promega Corp., Fitchburg, WI, USA) at 37°C for 75 min. In Milan, RNA was analyzed using the iScript™ one-step RT-PCR kit for probes (Bio-Rad), with RT-PCR performed in multiplexed reactions with a normalizing internal control (36B4) by use of the CFX384 real time system (Bio-Rad). Thermal cycling was initiated with an incubation at 50°C for 10 min (RNA retrotranscription), followed by 5 min at 95°C (TaqMan polymerase activation) and 39 reaction cycles with 10 s at 95°C and 30 s at 60°C. In Nijmegen, RT-PCR was performed with the CFX96 real time system (Bio-Rad). Prior to analysis of the relative expression of Nr3c1, Nr3c2 and Fkbp5, it was evaluated whether Rn18S, Gapdh, or Hprt1 would be the best internal control gene (also see Derks et al., 2008). Gapdh expression was found to be unaffected by our experimental design in all brain areas, and was therefore used as the internal control for this study. Furthermore, all primer pairs were tested for reaction efficiency. For the reactions a total volume of 25 μl of buffer solution was used containing 5 μl of template cDNA, 12.5 μl Power SYBR Green Master mix (Applied Biosystems, Foster City, CA, USA), 1.5 μl Rnase-free water and 0.6 μM of each primer. The cycling protocol started with 10 min at 95°C, followed by 39 reaction cycles with 15 s at 95°C and 1 min at 60°C. The procedure was concluded with a melting curve protocol, from 65 to 95°C, measuring fluorescence every 0.5°C, to control for product specificity. Primers and probe sequences used were purchased from Eurofins MWG-Operon (Ebersberg, Germany) and Biolegio (Nijmegen, Netherlands). All RT-PCR analyses were carried out in triplicate, with newly synthesized cDNA. Relative target gene expression was calculated by the 2^−ΔCt^ method (Schmittgen and Livak, 2008). The following primer and probe sequences were used; *Nr3c1*-Fw: 5′-GAAAAGCCATCGTCAAAA-GGG-3′, *Nr3c1*-Rev: 5′-TGGAAGCAGTAGGTAAGGAGA-3′, *Nr3c1*-Probe: 5′-AGCTTTGTCA-GTTGGTAAAACCGTTGC-3′, *Nr3c2*-Fw: 5′-TCGCTTTGAGTTGGAGATCG-3′, *Nr3c2*-Rev: 5′-ACGAATTGAAGGCTGATCTGG-3′, *Nr3c2*-Probe: 5′-AGTCTGCCATGTATGAACTGTGCCA-3′, *Fkbp5*-Fw: 5′-GAACCCAATGCTGAGCT-TATG-3′, *Fkbp5-Rev:* 5′-ATGTACTTGCCTCCCT-TGAAG-3′, *Fkbp5*-Probe: 5′-TGTCCATCTCCCAGGATTCTTTGGC-3′, *36B4*-Fw: 5′-TTCCCAC-TGGCTGAAAAGGT-3′, *36B4*-Rev: 5′-CGCAGCCGCAAATGC-3′, *36B4*-Probe: 5′-AAGGCCTT-CCTGGCCGATCCATC, *Gapdh*-Fw: 5′-GGTGTGAACGGATTTG-GC-3′, *Gapdh*-Rev: 5′-CTGG-AAGATGGTGATGGGTT-3′.

### Statistical analysis

All statistical tests have been carried out using SPSS (version 20, IBM corporation, Armonk, NY, USA). The results are presented as the mean with the standard error of the mean (SEM). The RT-PCR 2^−ΔCt^ data have been normalized to the average of the MS0-wild-type group and have been examined with factorial ANOVA. If a significant main effect (“genotype,” “ELS”) or interaction (“genotype × ELS”) was found, appropriate *a posteriori* tests were performed (One-Way ANOVA and independent samples *t*-test). Statistical significance was set at *p* < 0.05.

## Results

### GR mRNA levels

GR mRNA levels were found to be significantly affected by the interaction of ELS and 5-HTT genotype in the dorsal mPFC [*F*_(2, 31)_ = 7.08, *p* < 0.01] and dorsal hippocampus [*F*_(2, 33)_ = 4.57, *p* < 0.05]. In the dorsal hippocampus, a significant main effect of 5-HTT genotype [*F*_(2, 33)_ = 4.57, *p* < 0.05] was found in addition.

**Figure 1:**
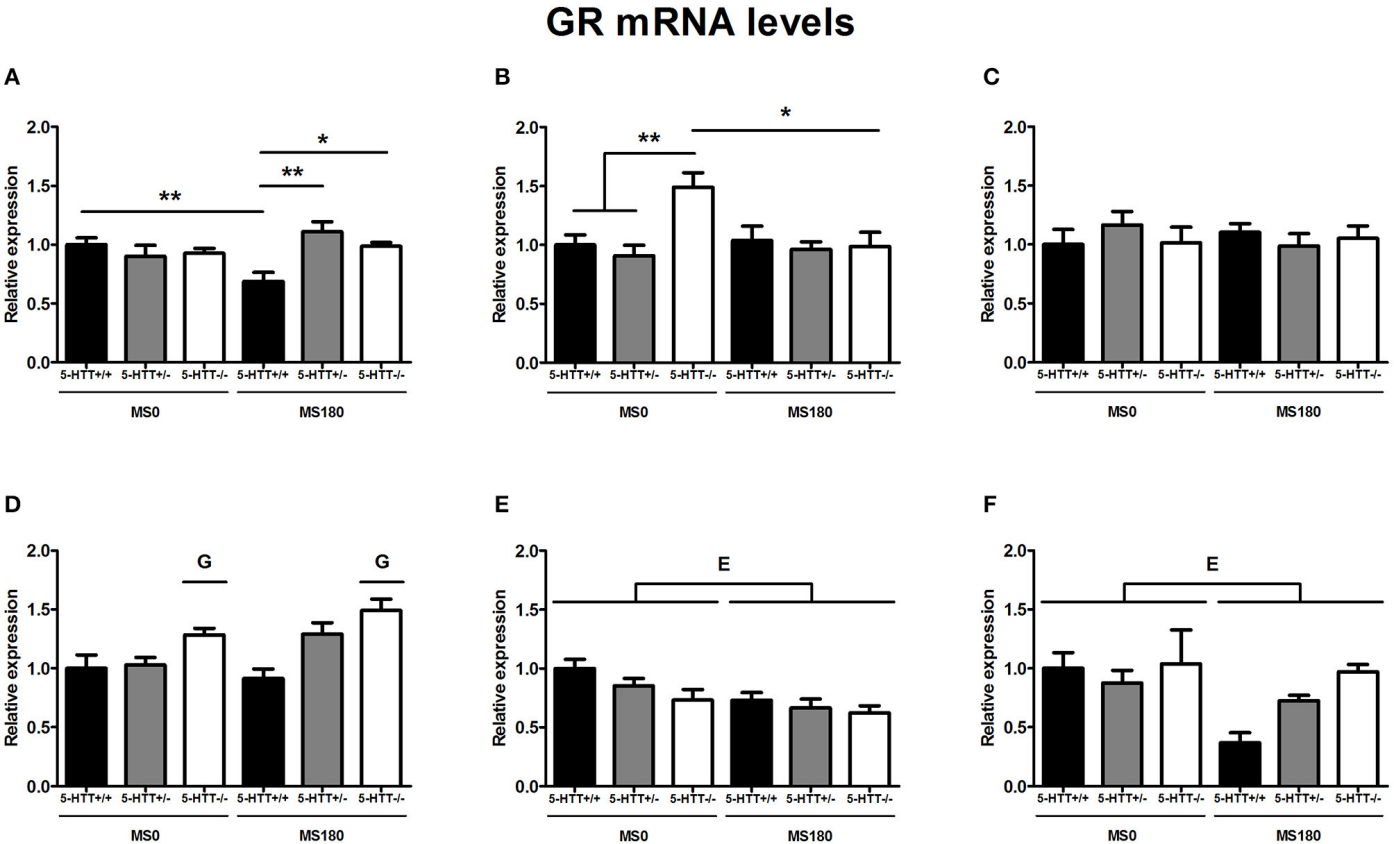
**Glucocorticoid receptor (GR) mRNA levels in the dorsal medial prefrontal cortex (mPFC) (A) dorsal hippocampus (B) central amygdala (C) ventral mPFC (D) ventral hippocampus (E) and the anterodorsal bed nucleus of the stria terminalis (BNST) (F) of serotonin transporter (5-HTT) homozygous knockout (5-HTT^−/−^), heterozygous knockout (5-HTT^+/−^), and wild-type (5-HTT^+/+^) rats exposed to daily 3 h separations (MS180) or a control treatment (MS0)**. GR mRNA levels in the dorsal mPFC and dorsal hippocampus were found to be affected by a significant interaction of early life stress (ELS) and 5-HTT genotype (*p* < 0.01, *p* < 0.05, respectively), with asterisks indicating significant pairwise comparisons (^*^p < 0.05 and ^**^p < 0.01). Furthermore, GR mRNA levels were affected by independent effects of 5-HTT genotype (G, *p* < 0.05) in the ventral mPFC and ELS (E, *p* < 0.05) in the ventral hippocampus and anterodorsal BNST. *Post-hoc* analysis revealed GR mRNA levels in the ventral mPFC to be higher for 5-HTT^−/−^ rats compared to 5-HTT^+/+^ rats (*p* < 0.001). Data were normalized to the average of the MS0-5-HTT^+/+^ group.

The exposure to ELS selectively decreased GR expression in the dorsal mPFC of 5-HTT^+/+^ rats (*p* < 0.01), leading to significantly lower GR mRNA levels for 5-HTT^+/+^ rats in comparison to 5-HTT^+/−^ (*p* < 0.01) and 5-HTT^−/−^ rats within the MS180 group (*p* < 0.05) (Figure 1A). In the dorsal hippocampus, 5-HTT^−/−^ rats displayed higher GR mRNA levels compared to 5-HTT^+/−^ and 5-HTT^+/+^ rats within the MS0 group (*p* < 0.01), but this effect of 5-HTT deficiency was not present in the case of ELS exposure (MS180 group) (Figure 1B).

Further, GR mRNA levels in the ventral hippocampus [*F*_(1, 30)_ = 9.62, *p* < 0.01] (Figure 1E) and the anterodorsal BNST [*F*_(1, 26)_ = 5.92, *p* < 0.05] (Figure 1F) were affected by a main effect of ELS (MS0 > MS180), while ventral mPFC GR mRNA levels were found to be affected by a main effect of 5-HTT genotype [*F*_(2, 29)_ = 11.62, *p* < 0.001], leading to higher GR expression in the ventral mPFC of 5-HTT^−/−^ rats in comparison to 5-HTT^+/+^ rats (*p* < 0.001) (Figure 1D). The GR mRNA levels in the central amygdala were not found to be affected by ELS, 5-HTT genotype or their interaction (Figure 1C).

### MR mRNA levels

MR mRNA levels in the dorsal hippocampus [*F*_(2, 26)_ = 14.09, *p* < 0.001] as well as the ventral hippocampus [*F*_(2, 29)_ = 7.10, *p* < 0.01] were found to be significantly affected by the interaction of ELS and 5-HTT genotype. These interaction effects were found together with main effects of ELS [*F*_(1, 29)_ = 11.59, *p* < 0.01] and 5-HTT genotype [*F*_(2, 29)_ = 26.92, *p* < 0.001] in the ventral hippocampus and a main effect of 5-HTT genotype [*F*_(2, 26)_ = 9.87, *p* < 0.01] in the dorsal hippocampus.

**Figure 2:**
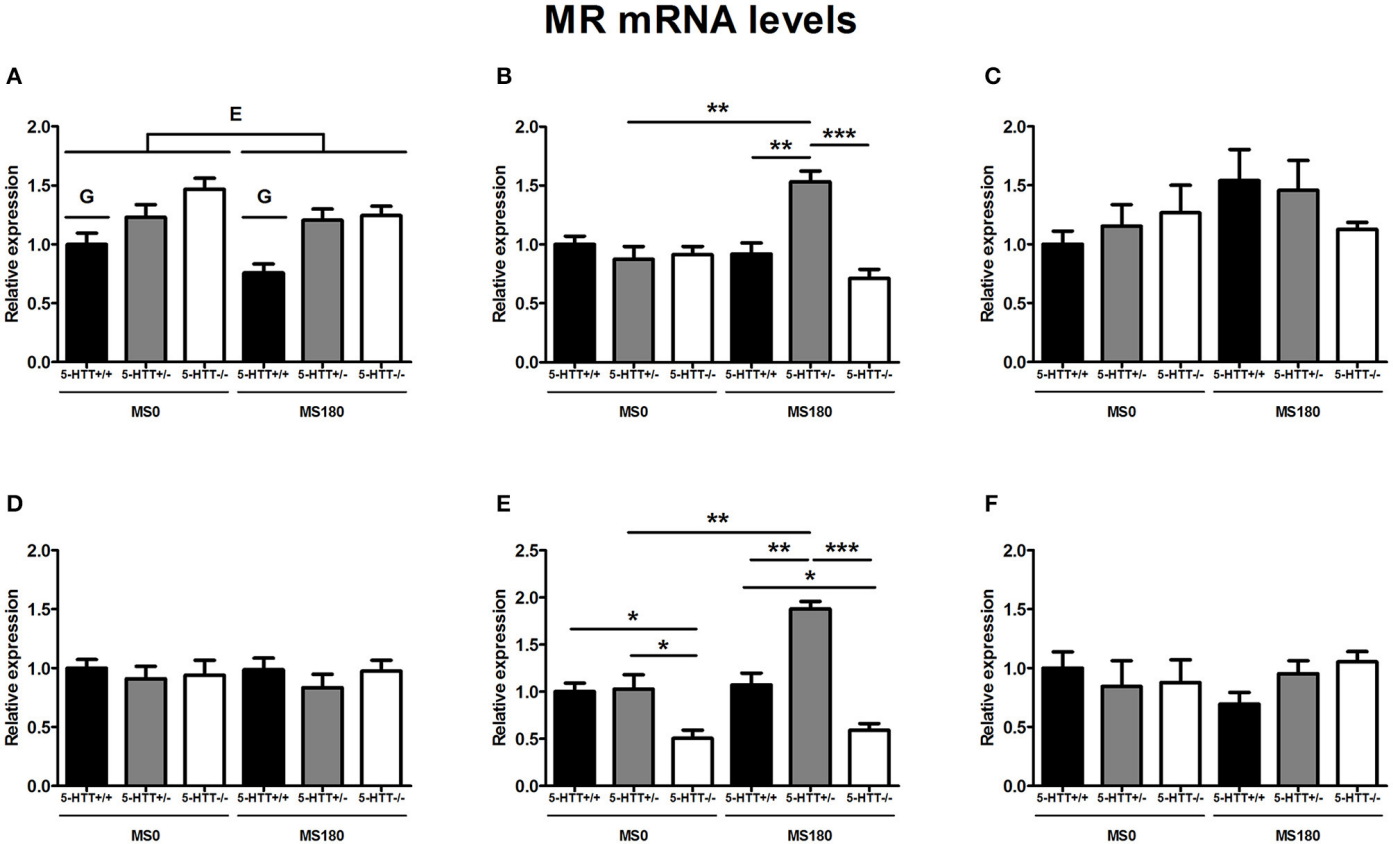
**Mineralocorticoid (MR) mRNA levels in the dorsal medial prefrontal cortex (mPFC) (A) dorsal hippocampus (B) central amygdala (C) ventral mPFC (D) ventral hippocampus (E) and the anterodorsal bed nucleus of the stria terminalis (F) of serotonin transporter (5-HTT) homozygous knockout (5-HTT^−/−^), heterozygous knockout (5-HTT^+/−^), and wild-type (5-HTT^+/+^) rats exposed to daily 3 h separations (MS180) or a control treatment (MS0)**. MR mRNA levels in the dorsal and ventral hippocampus were found to be affected by a significant interaction of early life stress (ELS) and 5-HTT genotype (*p* < 0.001, *p* < 0.01, respectively), with asterisks indicating significant pairwise comparisons (^*^p < 0.05, ^**^p < 0.01 and ^***^p < 0.001). Furthermore, MR mRNA levels were affected by independent effects of 5-HTT genotype (G, *p* < 0.001) and ELS (E, *p* < 0.05) in the dorsal mPFC. *Post-hoc* analysis revealed MR mRNA levels in the dorsal mPFC to be higher for both 5-HTT^+/−^ and 5-HTT^−/−^ rats compared to 5-HTT^+/+^ rats (*p* < 0.01 and *p* < 0.001, respectively). Data were normalized to the average of the MS0-5-HTT^+/+^ group.

For the dorsal hippocampus, MR mRNA levels were selectively increased for 5-HTT^+/−^ rats due to exposure to ELS (*p* < 0.01), leading to significantly higher MR expression for 5-HTT^+/−^ rats compared to 5-HTT^+/+^ (*p* < 0.01) and 5-HTT^−/−^ rats (*p* < 0.001) in the MS180 group (Figure 2B). In the ventral hippocampus, MR mRNA levels were significantly lower for 5-HTT^−/−^ rats in comparison to 5-HTT^+/+^ (*p* < 0.05) and 5-HTT^+/−^ rats (*p* < 0.05). The exposure to ELS led to a selective increase of MR expression for 5-HTT^+/−^ rats (*p* < 0.01), leading to significantly higher MR mRNA levels for 5-HTT^+/−^ rats compared to 5-HTT^+/+^ rats within the MS180 group (*p* < 0.01) and further increasing the significantly lower MR expression in the ventral hippocampus of 5-HTT^−/−^ rats compared to 5-HTT^+/−^ rats (*p* < 0.001) (Figure 2E).

In the dorsal mPFC, MR mRNA levels were found to be affected by main effects of both ELS [*F*_(1, 32)_ = 4.26, *p* < 0.05] and 5-HTT genotype [*F*_(2, 32)_ = 13.01, *p* < 0.001]. The exposure to ELS led to a decrease in MR expression (MS0 > MS180), while 5-HTT^+/−^, and 5-HTT^−/−^ rats were both found to display higher MR mRNA levels than 5-HTT^+/+^ rats (*p* < 0.01 and *p* < 0.001, respectively) (Figure 2A).

In the central amygdala, ventral mPFC, and anterodorsal BNST MR mRNA levels were not found to be affected by ELS, 5-HTT genotype or their interaction (Figures 2C,D,F).

### FKBP5 mRNA levels

FKBP5 mRNA levels were found to be significantly affected by the interaction of ELS and 5-HTT genotype in the dorsal mPFC [*F*_(2, 31)_ = 7.08, *p* < 0.01] and ventral mPFC [*F*_(2, 33)_ = 4.57, *p* < 0.05]. In addition, main effects of ELS in the dorsal mPFC [*F*_(1, 29)_ = 8.87, *p* < 0.01] and 5-HTT genotype in the ventral mPFC [*F*_(2, 29)_ = 7.89, *p* < 0.01] were identified.

**Figure 3:**
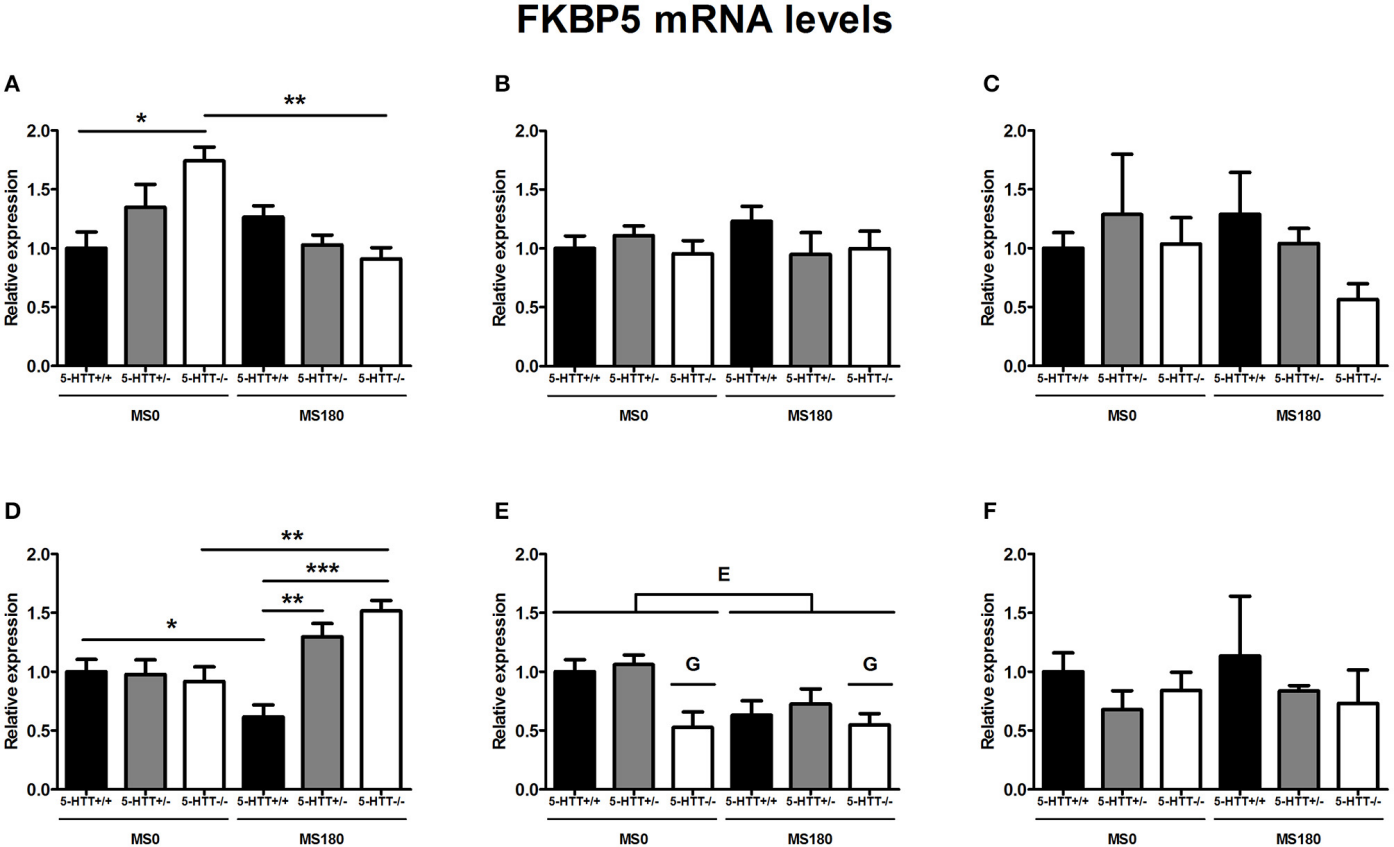
**FK506-binding protein 51 (FKBP5) mRNA levels in the dorsal medial prefrontal cortex (mPFC) (A) dorsal hippocampus (B) central amygdala (C) ventral mPFC (D) ventral hippocampus (E) and the anterodorsal bed nucleus of the stria terminalis (F) of serotonin transporter (5-HTT) homozygous knockout (5-HTT^−/−^), heterozygous knockout (5-HTT^+/−^), and wild-type (5-HTT^+/+^) rats exposed to daily 3 h separations (MS180) or a control treatment (MS0)**. FKBP5 mRNA levels in the dorsal and the ventral mPFC were found to be affected by a significant interaction of early life stress (ELS) and 5-HTT genotype (*p* < 0.01, *p* < 0.05, respectively), with asterisks indicating significant pairwise comparisons (^*^p < 0.05, ^**^p < 0.01 and ^***^p < 0.001). Furthermore, FKBP5 mRNA levels were affected by independent effects of 5-HTT genotype (G, *p* < 0.05) and ELS (E, *p* < 0.05) in the ventral hippocampus. *Post-hoc* analysis revealed FKBP5 mRNA levels in the ventral hippocampus to be lower for 5-HTT^−/−^ rats compared to 5-HTT^+/−^ rats (*p* < 0.05). Data were normalized to the average of the MS0-5-HTT^+/+^ group.

For the dorsal mPFC, within the MS0 group 5-HTT^−/−^ rats displayed increased FKBP5 mRNA levels compared to 5-HTT^+/+^ rats (*p* < 0.05). After exposure to ELS however, FKBP5 expression decreased only in 5-HTT^−/−^ rats (*p* < 0.01), and no difference was found between 5-HTT^−/−^ and 5-HTT^+/+^ rats (Figure 3A). In contrast, FKBP5 mRNA levels in the ventral mPFC were found to be increased for 5-HTT^−/−^ rats (*p* < 0.01), but decreased for 5-HTT^+/+^ rats (*p* < 0.05), after exposure to ELS. Therefore, within the MS180 group 5-HTT^−/−^ and 5-HTT^+/−^ rats showed significantly higher FKBP5 mRNA levels compared to 5-HTT^+/+^ rats (*p* < 0.001 and *p* < 0.01, respectively) (Figure 3D).

Further, FKBP5 mRNA levels in the ventral hippocampus were found to be significantly affected by main effects of both ELS [*F*_(1, 26)_ = 5.85, *p* < 0.05] and 5-HTT genotype [*F*_(2, 26)_ = 5.09, *p* < 0.05]. The exposure to ELS led to a decrease in FKBP5 expression (MS0 > MS180), while 5-HTT^−/−^ rats were found to display significantly lower FKBP5 mRNA levels than 5-HTT^+/−^ rats (*p* < 0.05) (Figure 3E).

In the dorsal hippocampus, central amygdala and anterodorsal BNST FKBP5 mRNA levels were not found to be affected by ELS, 5-HTT genotype or their interaction (Figures 3B,C,F).

## Discussion

In this study, we found that ELS exposure differentially induced adaptations of GR, MR, and FKBP5 mRNA levels at extra-hypothalamic sites in adult 5-HTT^+/+^, 5-HTT^+/−^, and 5-HTT^−/−^ rats. It remains to be experimentally determined how these findings could be related to the altered activity of the HPA-axis (van der Doelen et al., 2014) and stress coping behavior (van der Doelen et al., 2013) that we previously reported of our animal model of ELS × 5-HTTLPR interaction. Moreover, as we and others have recently shown (Daskalakis et al., 2012; van der Doelen et al., 2013; Santarelli et al., 2014), the exposure to stress does not necessarily need to have negative consequences, but can rather induce phenotypic changes that have adaptive value in coping with later life stressors. As such, these findings support the recently postulated match/mismatch hypothesis (Champagne et al., 2009; Nederhof and Schmidt, 2012), which states that individuals can use the experience of past stressors to adaptively respond to future challenges (predictive adaptive response) (Gluckman et al., 2007). The match/mismatch hypothesis predicts that such phenotypic changes are beneficial when the individuals face similar environments later in life (match), but could be maladaptive if the environment changes significantly (mismatch). The match/mismatch hypothesis is not compatible with a deterministic view as it implies that the prior life history and the specific demands of current/future stressors (Myers et al., 2014) will dictate whether a given phenotype is adaptive or maladaptive. Therefore, it remains to be elucidated under which specific conditions the differential expression of GR, MR, and FKBP5 mRNA levels in our animal model of ELS × 5-HTTLPR interaction will turn out to be adaptive or maladaptive. For now, we will discuss the current findings from different perspectives in the following sections.

### ELS × 5-HTT genotype: anatomical specificities

Interestingly, the effects of ELS × 5-HTT genotype interaction showed a gene-dependent anatomical distinction, with MR mRNA levels affected only in the hippocampus, FKBP5 mRNA only in the mPFC, and GR mRNA only in the dorsal regions of the mPFC and hippocampus. In contrast to these higher order/processing areas of the limbic system, for the extended amygdala areas (central amygdala and anterodorsal BNST) we found that ELS exposure, irrespective of genotype, affected only GR mRNA levels in the anterodorsal BNST. The influence of ELS × 5-HTT genotype interaction was therefore clearly stronger in the mPFC and hippocampus compared to the central amygdala and anterodorsal BNST.

Another interesting observation is that the effects of ELS and 5-HTT genotype on GR, MR, and FKBP5 expression were markedly different for dorsal vs. ventral subregions of both mPFC and hippocampus. The dorsal and ventral mPFC punches in this study correspond to the functionally distinct prelimbic and infralimbic cortices, which have been linked to opposite roles in the control of fear responses (Sotres-Bayon and Quirk, 2010). Here, we found that the dorsal and ventral mPFC of 5-HTT^−/−^ rats also showed opposite regulation of FKBP5 mRNA levels after ELS exposure. Regarding the hippocampus, it has been argued that the dorsal regions perform primarily cognitive functions, while ventral subregions are related to stress, emotion, and affect (Fanselow and Dong, 2010). In our model, we found that the ventral hippocampus showed more alterations in GR, MR, and FKBP5 mRNA levels by the interaction of ELS, and 5-HTT genotype than the dorsal hippocampus, although the latter was certainly not unaffected. The physiological and behavioral consequences of distinct programming of dorsal vs. ventral mPFC and hippocampus subregions should be investigated further, both in our and other animal models of stress-related diseases.

### ELS × 5-HTT genotype: GR/MR balance

The balance between GR and MR functioning has been proposed to be central to stress-related psychopathology, as GR and MR serve such complementary glucocorticoid functions during the stress response (De Kloet et al., 2005). For instance, hippocampal GR is involved in the consolidation of emotional memory and disinhibition of the HPA-axis (Roozendaal and McGaugh, 1997; De Kloet et al., 1998), while MR is important for maintaining basal HPA activity and memory retrieval (De Kloet et al., 1998; Dorey et al., 2011). Essential to this functional segregation is the 10-fold higher affinity of glucocorticoid binding by MRs compared to GRs. Low basal corticosterone levels therefore predominantly occupy MR, while GR is activated by stress-induced as well as ultradian peaks of glucocorticoid levels (De Kloet et al., 1998; Lightman et al., 2008). To explore the relevance of a functional GR/MR disbalance in ELS × 5-HTTLPR interactions, we will discuss our findings here in terms of the balance between programmed expression levels of GR and MR.

In addition to the selective effects of ELS on different 5-HTT genotypes, the exposure to ELS was found to lead in all genotypes to decreased MR mRNA levels in the dorsal mPFC, decreased GR mRNA levels in the anterodorsal BNST and decreased GR and FKBP5 mRNA levels in the ventral hippocampus. As FKBP5 has mainly been characterized as an inhibitory co-chaperone for GR and not MR (Binder, 2009), it is assumed at present that FKBP5 chiefly regulates GR function. In the ventral hippocampus, ELS exposure decreased both GR and FKBP5 mRNA levels, and it can therefore be assumed that GR/MR balance in the ventral hippocampus is not affected by a general effect of ELS.

For the anterodorsal BNST, it seems that ELS exposure only leads to a decrease in GR mRNA in 5-HTT^+/+^ rats, although the ELS × 5-HTT genotype interaction was not found to significantly affect anterodorsal BNST GR mRNA levels. In the 5-HTT^+/+^ rats, ELS exposure furthermore selectively decreased ventral mPFC FKBP5, and dorsal mPFC GR mRNA levels. The former could affect local GR/MR balance, but the latter would be expected to counterbalance the general ELS effect of decreased dorsal mPFC MR mRNA levels. Therefore, after ELS exposure, transcriptional GR/MR balance in 5-HTT^+/+^ rats only seems to be affected in the anterodorsal BNST and the ventral mPFC, with a relative increase in MR over GR transcription in the anterodorsal BNST and the opposite in the ventral mPFC.

For 5-HTT^+/−^ rats, ELS exposure led to increased MR mRNA levels in both the dorsal and ventral hippocampus, while increased dorsal mPFC MR expression is present both under control conditions and after ELS exposure in 5-HTT^+/−^ rats. The latter should compensate for the general ELS effect of decreased dorsal mPFC MR mRNA levels. As such, after ELS exposure, GR/MR disbalance in 5-HTT^+/−^ seems to be limited to the hippocampus, with a relative increase in MR over GR transcription.

For 5-HTT^−/−^ rats, baseline observations included increased GR mRNA in the dorsal hippocampus and ventral mPFC, decreased FKBP5 and MR mRNA in the ventral hippocampus, and increased FKBP5, and MR mRNA in the dorsal mPFC. The latter, just as for 5-HTT^+/−^ rats, would be expected to compensate for the general ELS effect of decreased dorsal mPFC MR mRNA levels. Further, after exposure to ELS, the increased GR, and FKBP5 mRNA levels in respectively, the dorsal hippocampus and mPFC were no longer present. In addition, ELS exposure led to an increase in ventral mPFC FKBP5 mRNA levels selectively in 5-HTT^−/−^ rats, which would be expected to counterbalance the increased ventral mPFC GR mRNA levels observed under control conditions. Therefore, transcriptional GR/MR balance in 5-HTT^−/−^ rats after ELS exposure seems to be only affected in the ventral hippocampus, by the decreased expression of both MR and FKBP5 in this area. In contrast to 5-HTT^+/−^ rats, this putatively disturbed hippocampal GR/MR balance would be predicted to lead to a relative increase of GR over MR function.

Overall, we observe a strong moderation by 5-HTT genotype of ELS-induced disbalances between GR and MR mRNA levels. We acknowledge that it remains to be empirically proven whether transcriptional GR/MR disbalances ultimately lead to functional GR/MR disbalance in our model, and, as the regulation of GR/MR/FKBP5 expression is highly dynamic, it remains to be determined how stable these transcriptional disbalances would be.

### ELS × 5-HTT genotype: possible functional consequences

As said above, the interaction of ELS and 5-HTT genotype resulted in differential adult expression patterns of GR, MR, and FKBP5 mRNA levels in the mPFC and hippocampus of 5-HTT^+/+^, 5-HTT^+/−^, and 5-HTT^−/−^ rats. From a genetic perspective, the 5-HTT^+/−^ rats can be considered as the best model for human 5-HTTLPR S-allele carriers (Homberg et al., 2007). Only in these rats, the exposure to ELS led to an increase of MR mRNA levels in the dorsal as well as ventral hippocampus. The hippocampal MR has previously been shown to be involved in the stress-induced switching between learning/coping strategies (Oitzl and De Kloet, 1992; Schwabe et al., 2009). Therefore, the ELS-induced elevation of hippocampal MR expression in 5-HTT^+/−^ rats could increase the use of habit-based learning strategies under stressful conditions (Schwabe et al., 2009). Furthermore, increased MR mRNA levels could increase the inhibitory regulation of the hippocampus on basal HPA activity (De Kloet et al., 1998). In 5-HTT^+/+^ rats, ELS led to decreased GR mRNA levels in the dorsal mPFC, and decreased FKBP5 mRNA levels in the ventral mPFC. These alterations could affect the feedback control exerted by the mPFC over HPA activity (McKlveen et al., 2013). Furthermore, given the inhibitory regulation of FKBP5 on GR activity (Binder, 2009), GR function could be relatively decreased in the dorsal mPFC, but increased in the ventral mPFC of 5-HTT^+/+^ rats exposed to ELS. This could result in an increased relative influence of stress-induced glucocorticoids on extinction vs. expression of fear memory (Gourley et al., 2009; Sotres-Bayon and Quirk, 2010). In contrast, ELS-exposed 5-HTT^−/−^ rats showed a downregulation of FKBP5 mRNA in the dorsal mPFC, and an upregulation of FKBP5 mRNA in the ventral mPFC. Possibly, these changes also lead to an opposite effect in the relative impact of stress-induced glucocorticoids on emotional memory processing compared to ELS-exposed 5-HTT^+/+^ rats. Interestingly, naïve 5-HTT^−/−^ rodents already display an impairment in fear extinction recall (Wellman et al., 2007; Nonkes et al., 2012).

### Translation of findings

As ELS and ELS × 5-HTT genotype interaction are associated with depression, we expected to find similar changes in the transcription of GR, MR, and FKBP5 as reported in clinical studies. In major depression, decreased MR mRNA in the anterior cingulate cortex (ACC) has been documented recently (Qi et al., 2013). On the basis of structure and function, the dorsal part of the ACC is thought to be homologous to the rodent prelimbic cortex (Uylings et al., 2003). In our study, a part of this cortical area was captured by the dorsal mPFC punches and we found indeed that ELS exposure led to decreased MR expression in these samples. However, 5-HTT deficiency was found to be associated with increased MR mRNA levels in this area. In the case of the hippocampus, depressed subjects have been reported to display decreased GR and MR mRNA levels (Webster et al., 2002; Klok et al., 2011a). In addition, a recent study found decreased MR mRNA selectively in the anterior hippocampus (Medina et al., 2013), which corresponds to the rodent ventral hippocampus (Fanselow and Dong, 2010). In our study, we found that ELS was indeed associated with decreased GR mRNA and 5-HTT knockout with decreased MR mRNA in the ventral hippocampus. However, the interaction of ELS and 5-HTT genotype resulted in an increase of MR mRNA in the ventral hippocampus in 5-HTT^+/−^ rats, which would not fit the increased risk for depression associated with the GxE interaction.

Exposure to childhood abuse has been associated with reduced hippocampal GR expression (McGowan et al., 2009), but this has not been reported (thus far) for childhood neglect. Indeed, childhood abuse is more frequently studied, in part due to the higher level of heterogeneity in cases of childhood neglect, although both types of maltreatment are convincingly associated with psychopathology (Teicher and Samson, 2013). We observed that the offspring that had been subjected to the MS paradigm showed a reduction in GR mRNA levels in the ventral hippocampus. Whereas MS is an established model for ELS exposure, it however is likely a better approximation of the human situation of childhood neglect compared to that of childhood abuse. In the laboratory setting, the mother rat is observed to be frequently away from the nest for periods of 20–25 min (Jans and Woodside, 1990; Francis et al., 2002). In seminaturalistic conditions, subordinate mothers are often forced to build their nests far from nutritional sources, and this environmental challenge has been reported to lead to periods of separation for 2–3 h (Calhoun, 1962; Meaney, 2001). Therefore, the daily 3 h separations are considered to be an ethologically relevant stressor for the offspring, which results in a deprivation of maternal care, and which has not been observed to lead to abusive behavior of the mother rat. Consequently, our finding that rats subjected to MS display reduced GR mRNA levels in the ventral hippocampus may indicate that this phenotype is not specific to childhood abuse, but may also apply to childhood neglect.

### Study limitations and comparison to rodent literature

There are some limitations of the current study that should be mentioned. First, we examined here the expression of GR, MR, and FKBP5 at the mRNA level, but do not complement this with reporting the levels of the corresponding proteins, the functional end products of the genes. Secondly, we have used real-time PCR in combination with biopsy punching as a quantitative approach instead of *in situ* hybridization. Due to this approach we had to make a selection of brain areas and lose anatomical resolution. We could therefore for instance not assess mRNA levels in separate cortical layers, or in the hippocampal subregions, e.g., the Cornu Ammonis (CA) areas and dentate gyrus (DG). Thirdly, to limit the amount of social stress, the rats were housed with their littermates after weaning. The rats were therefore housed in same-treatment groups (MS0/MS180), potentially constituting differential living environments on top of the early life treatment, which together may have led to the observed adaptations in gene expression.

MS has been shown before to lead to increased GR-immunoreactivity (GR-ir) in the CA1 region, but not the DG of the dorsal hippocampus of Sprague-Dawley rats (Biagini et al., 1998). In this rat strain, decreased total hippocampal GR mRNA levels have also been reported (Maniam and Morris, 2010). In Long-Evans rats, MS has been observed to lead to decreased GR mRNA and increased MR mRNA levels in the dorsal hippocampus (Ladd et al., 2004, 2005). Furthermore, Long-Evans rats that have experienced low quality of maternal care as pups (low levels of licking and grooming), a different but related model of early life adversity, display decreased hippocampal GR-ir (total hippocampus) and GR mRNA levels (dorsal hippocampus) (Liu et al., 1997; Weaver et al., 2004). In all of these studies, the ventral hippocampus has not been studied in isolation for the expression of GR and MR. Here, we have found that MS induces a decrease of GR mRNA levels in the ventral hippocampus of all 5-HTT genotypes. We observed that not 5-HTT^+/+^ rats, but the putatively more stress-sensitive 5-HTT^+/−^, and 5-HTT^−/−^ rats displayed increased MR mRNA and decreased GR mRNA in the dorsal hippocampus, respectively. Our rats have a Wistar background, and given the present literature, it is possible that the genetic background of rat strains modulates the effects of ELS exposure (MS) on the expression of GR and MR (see also Ellenbroek and Cools, 2000). Previously, Wistar rats exposed to MS have been shown to exhibit decreased GR-ir of the total hippocampus (Aisa et al., 2007), while others did not find alterations of GR and MR mRNA levels (Wang et al., 2013), and unaltered GR-ir in the dorsal hippocampus (Renard et al., 2010; Vivinetto et al., 2013).

## Conclusions

In conclusion, we report here that ELS and 5-HTT genotype interactively affect the expression of GR, MR, and FKBP5 in a brain area-specific way, with further distinction for dorsal vs. ventral subregions of the mPFC and hippocampus. These results could be a starting point for studies aiming to elucidate the role of altered extra-hypothalamic glucocorticoid signaling in the depressogenic interaction of ELS and 5-HTTLPR in humans.

## Author contributions

Rick H. A. van der Doelen: experimental design, animal experiments, tissue collection, data analysis and writing; Francesca Calabrese: RNA isolation, real-time PCR, data analysis, writing; Gianluigi Guidotti: RNA isolation, real-time PCR, data analysis; Bram Geenen: real-time PCR; Marco A. Riva: overall discussion, writing; Tamás Kozicz: project design, experimental design, funding, writing; Judith R. Homberg: project design, experimental design, funding, writing. All authors have approved the final manuscript.

## Funding

Funding for this study was provided by an ALW grant from the Netherlands Organisation for Scientific Research (NW) to Judith R. Homberg and Tamás Kozicz (819.02.022).

### Conflict of interest statement

The authors declare that the research was conducted in the absence of any commercial or financial relationships that could be construed as a potential conflict of interest.
