# Supplementary material for: Early life stress and serotonin transporter gene variation interact to affect the transcription of the glucocorticoid and mineralocorticoid receptors, and the co-chaperone FKBP5, in the adult rat brain
Source: Front Behav Neurosci. 2014 Oct 13;8:355. doi: 10.3389/fnbeh.2014.00355 (PMC4195371; doi:10.3389/fnbeh.2014.00355)

Supplementary material Van der Doelen *et al*., 2014

Example images of punched rat brain sections

Medial Prefrontal Cortex

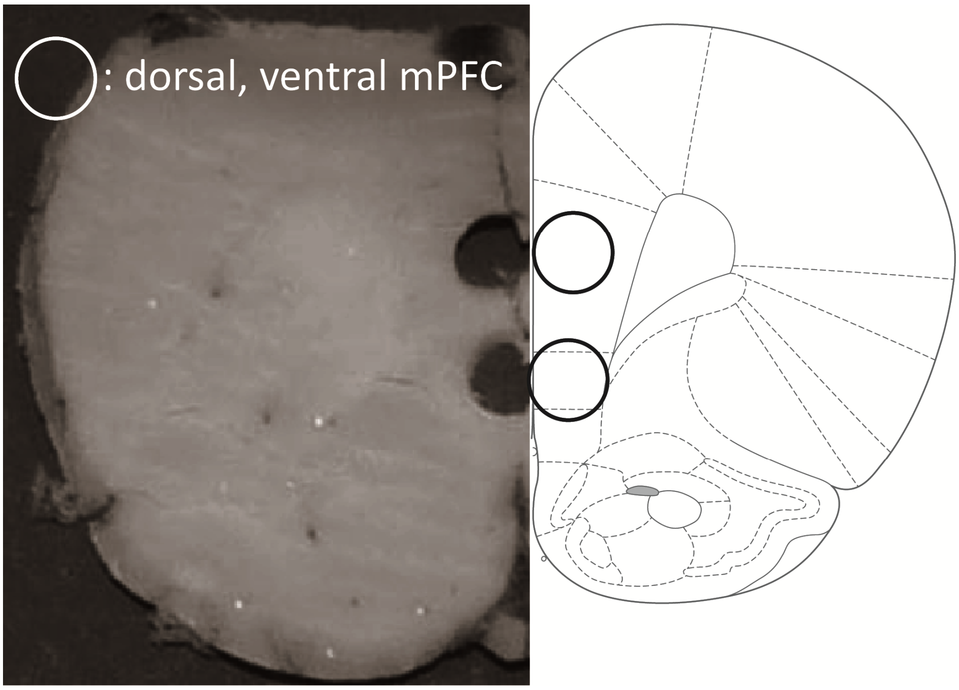

Anterodorsal bed nucleus of the stria terminalis

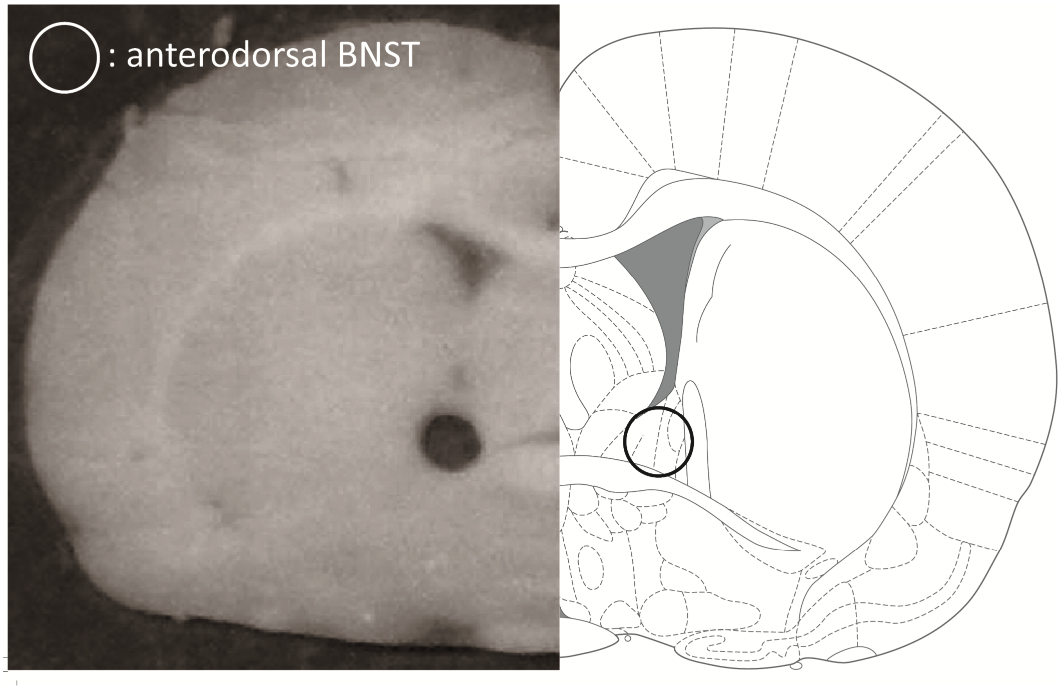

Dorsal Hippocampus & Central Amygdala

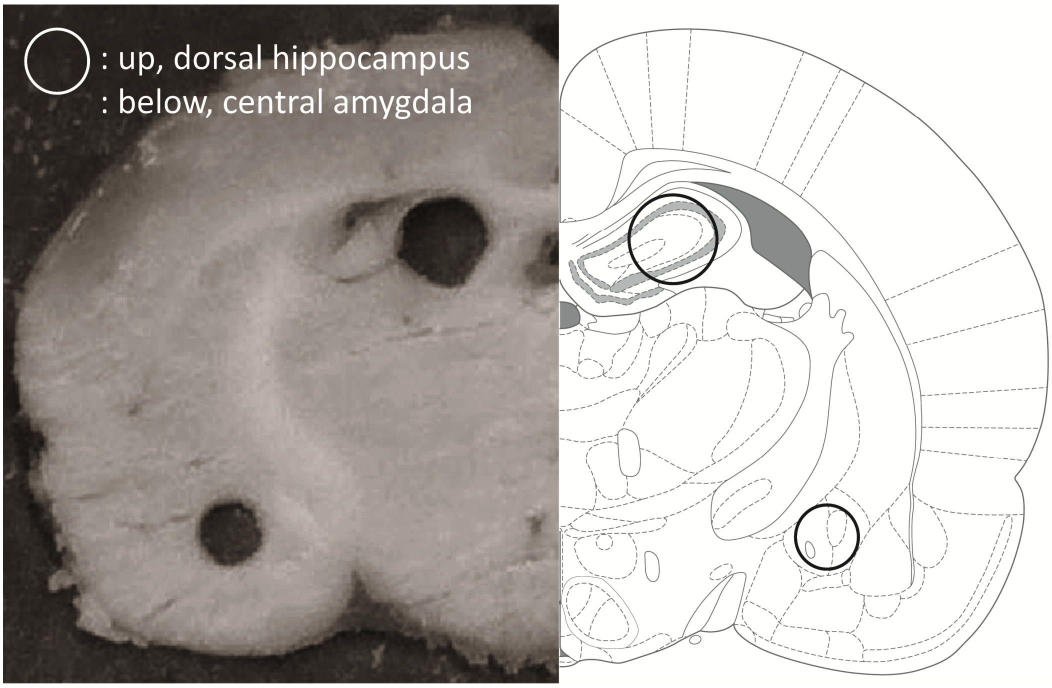

Ventral Hippocampus

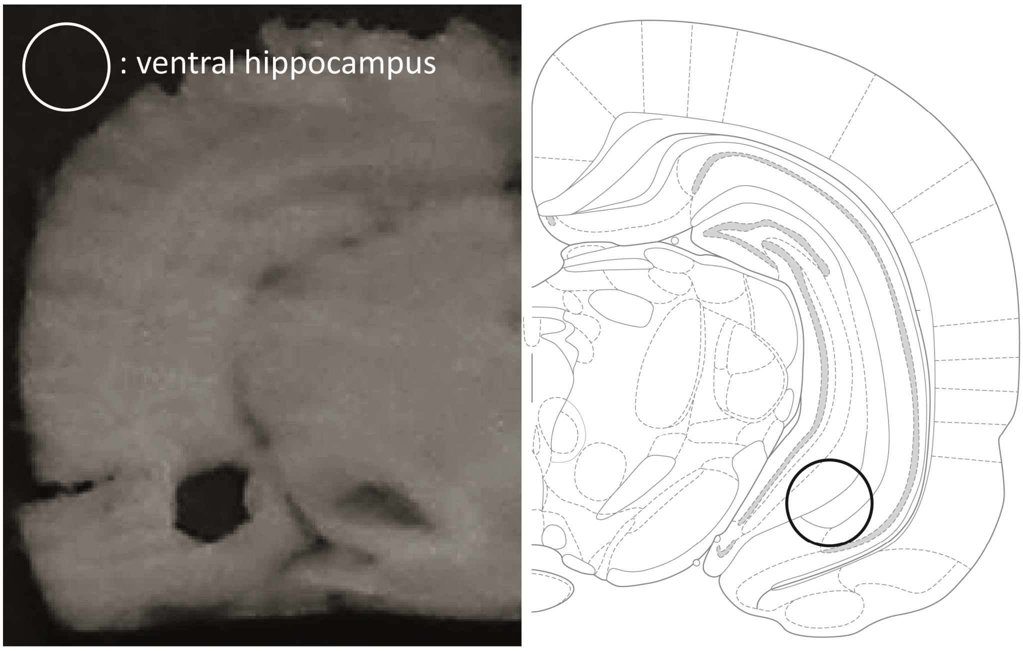

Supplement: Supplementary file 1 [file DataSheet1.PDF]
