# Supplementary material for: Early life stress and serotonin transporter gene variation interact to affect the transcription of the glucocorticoid and mineralocorticoid receptors, and the co-chaperone FKBP5, in the adult rat brain
Source: Front Behav Neurosci. 2014 Oct 13;8:355. doi: 10.3389/fnbeh.2014.00355 (PMC4195371; doi:10.3389/fnbeh.2014.00355)
Supplement: Supplementary file 2 [file DataSheet2.PDF]

Van der Doelen et al.: Early life stress and serotonin transporter gene variation interact to affect the transcription of the glucocorticoid and mineralocorticoid receptor and the co-chaperone FKBP5 in the adult rat brain.

# Supplementary material Van der Doelen *et al.*, 2014

## Group sizes of analyzed variables

| **dmPFC** | MS0 | | | MS180 | | |
|---|---|---|---|---|---|---|
| | 5-HTT$^{+/+}$ | 5-HTT$^{+/-}$ | 5-HTT$^{-/-}$ | 5-HTT$^{+/+}$ | 5-HTT$^{+/-}$ | 5-HTT$^{-/-}$ |
| GR | 7 | 7 | 5 | 6 | 7 | 5 |
| MR | 7 | 7 | 6 | 7 | 7 | 4 |
| FKBP5 | 6 | 5 | 5 | 7 | 7 | 5 |
| **vmPFC** | MS0 | | | MS180 | | |
| | 5-HTT$^{+/+}$ | 5-HTT$^{+/-}$ | 5-HTT$^{-/-}$ | 5-HTT$^{+/+}$ | 5-HTT$^{+/-}$ | 5-HTT$^{-/-}$ |
| GR | 7 | 6 | 5 | 7 | 5 | 5 |
| MR | 8 | 6 | 6 | 7 | 5 | 5 |
| FKBP5 | 7 | 5 | 6 | 7 | 6 | 4 |
| **dHP** | MS0 | | | MS180 | | |
| | 5-HTT$^{+/+}$ | 5-HTT$^{+/-}$ | 5-HTT$^{-/-}$ | 5-HTT$^{+/+}$ | 5-HTT$^{+/-}$ | 5-HTT$^{-/-}$ |
| GR | 8 | 7 | 6 | 6 | 7 | 5 |
| MR | 6 | 6 | 6 | 5 | 5 | 4 |
| FKBP5 | 7 | 5 | 5 | 5 | 7 | 4 |
| **vHP** | MS0 | | | MS180 | | |
| | 5-HTT$^{+/+}$ | 5-HTT$^{+/-}$ | 5-HTT$^{-/-}$ | 5-HTT$^{+/+}$ | 5-HTT$^{+/-}$ | 5-HTT$^{-/-}$ |
| GR | 7 | 7 | 5 | 7 | 6 | 4 |
| MR | 6 | 8 | 6 | 6 | 5 | 4 |
| FKBP5 | 5 | 7 | 6 | 6 | 4 | 4 |

| **CeA** | MS0 | | | MS180 | | |
|---|---|---|---|---|---|---|
| | 5-HTT$^{+/+}$ | 5-HTT$^{+/-}$ | 5-HTT$^{-/-}$ | 5-HTT$^{+/+}$ | 5-HTT$^{+/-}$ | 5-HTT$^{-/-}$ |
| GR | 6 | 5 | 6 | 5 | 6 | 4 |
| MR | 6 | 5 | 6 | 5 | 6 | 4 |
| FKBP5 | 6 | 5 | 6 | 5 | 6 | 4 |
| **adBNST** | MS0 | | | MS180 | | |
| | 5-HTT$^{+/+}$ | 5-HTT$^{+/-}$ | 5-HTT$^{-/-}$ | 5-HTT$^{+/+}$ | 5-HTT$^{+/-}$ | 5-HTT$^{-/-}$ |
| GR | 8 | 6 | 4 | 5 | 6 | 3 |
| MR | 8 | 6 | 4 | 5 | 6 | 3 |
| FKBP5 | 8 | 6 | 4 | 5 | 6 | 3 |
